# A Narrative Review of Childhood Myopia Management Across the NHS and Private Sector in the UK

**DOI:** 10.7759/cureus.104805

**Published:** 2026-03-07

**Authors:** Hammaad Khalid, Mishelle Abbasi, Mohamed Mohyudin

**Affiliations:** 1 Medicine, Leeds Teaching Hospitals NHS Trust, Leeds, GBR; 2 Paediatrics, Airedale Hospital, Leeds, GBR; 3 Ophthalmology, Calderdale Royal Hospital, Halifax, GBR

**Keywords:** childhood myopia, myopia, myopia prevention and control, myopia progression, myopia treatment, paediatric myopia, paediatrics

## Abstract

Childhood myopia is a growing concern in the United Kingdom (UK) and is associated with long-term ocular risks. Early interventions can slow progression and reduce complications. The National Health Service (NHS) manages myopia through visual correction, monitoring and lifestyle advice. Interventions such as dual-focus lenses, orthokeratology and low-dose atropine are typically accessed through private care in the UK. A PubMed search conducted in December 2025 identified 30 studies on evaluating childhood myopia management published between January 2000 and December 2025, with an age limit of 0-18 years and in English. These studies were reviewed using a narrative comparative approach. Studies show these interventions significantly reduce disease progression and axial elongation, supporting their role in myopia control. National guidelines and funded care pathways could help bridge the gap between private and NHS care, ensuring equitable access and reducing the long-term burden of myopia in the UK.

## Introduction and background

Introduction

Myopia is also known as near-sightedness. This is a condition where it becomes difficult to visualise far objects due to the refractive error of the eye or due to the excessive growth in axial length of the eyeball [1]. Research showed that the prevalence of paediatric myopia has more than doubled in recent years, and the age of onset is now earlier in childhood [2]. Data demonstrates an increased incidence and earlier onset in white children in the United Kingdom (UK) with refractive change occurring between six and 13 years [2]. Estimates predict that by 2050, myopia could affect almost half the world’s population, with progression to high myopia (≥-6.00D) predicted to impact around 10% of the world’s population [3]. This is a public health concern since the earlier onset of myopia has been associated with faster disease progression and increased risks of developing complications such as glaucoma, retinal detachment, posterior subcapsular cataracts and myopic maculopathy [4,5].

Modelling studies have shown a relationship between increasing dioptres of myopia and lifetime risk of visual impairment, highlighting that each increase in dioptre is associated with increased risk of myopic progression [4]. Decision support tools based on absolute risk modelling may help clinicians communicate the long-term benefits of early myopic control to families.

Traditional management of myopia involves refractive correction with spectacles or contact lenses, which restores visual acuity but does not influence axial elongation. In contrast, myopia control interventions aim to slow ocular growth and reduce disease progression. These include pharmacological approaches such as low-dose atropine and optical interventions including orthokeratology and specialised multifocal or dual-focus contact lenses [6].

Within the UK, children with suspected myopia are typically assessed in community optometry services and referred to hospital eye services if atypical features, ocular pathology or rapid progression are identified. Whilst refractive correction is widely available within the NHS, most interventions specifically designed to slow myopia progression are not routinely commissioned and are therefore predominantly accessed through the private sector [7,8]. Given the increasing prevalence of paediatric myopia and the expanding range of interventions aimed at slowing disease progression, there is growing interest in strategies to mitigate its long-term visual and societal burden [9].

Methodology

A structured literature search was performed to identify relevant studies on childhood myopia management. This was conducted using PubMed in December 2025. A predefined set of search terms were used that included keywords such as “childhood myopia”, “myopia control”, “NHS”, “private optometry”, “low-dose atropine”, “orthokeratology” and “multifocal contact lenses”. Boolean operators were used to refine the search.

The review was conducted using transparent reporting principles inspired by a Preferred Reporting Items for Systematic Reviews and Meta-Analyses (PRISMA)-style study selection process; however, this was not a formal systematic review, so a formal PRISMA was not conducted.

Inclusion criteria were narrowed down to human studies published between January 2000 and December 2025, published in English with full free texts available, texts that focused on participants aged 0-18 years and publications that focused on the management or control of myopia. Exclusion criteria included non-human studies, editorials, conference abstracts, opinion pieces and non-myopia-related studies.

Titles and abstracts were screened for relevance, followed by a full-text review of the literature. A total of 30 studies were included in the final analysis.

**Figure 1:**
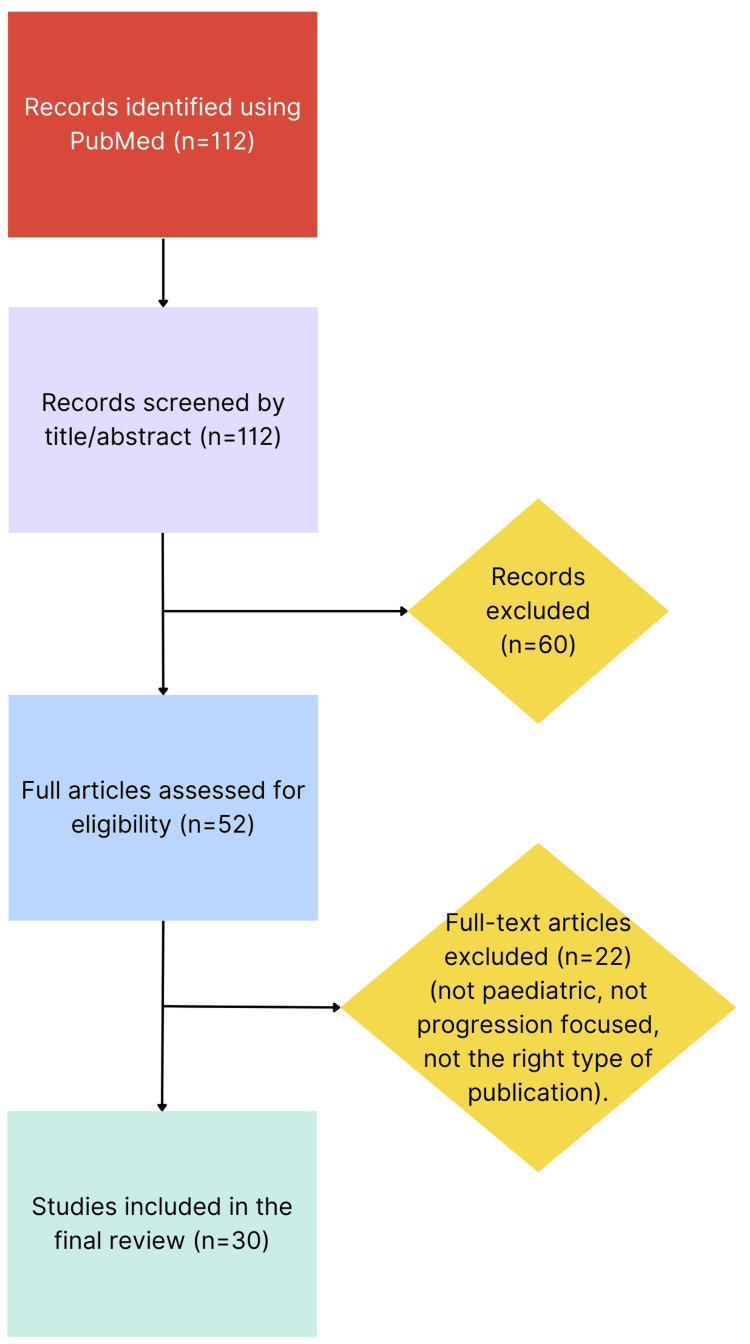
Study selection flowchart inspired by PRISMA PRISMA: Preferred Reporting Items for Systematic Reviews and Meta-Analyses

Included studies were organised descriptively using a thematic comparative approach and grouped by intervention type: optical, pharmacological and lifestyle strategies. This then allowed for comparisons to be made between the NHS and private services in the UK. Evidence strength was based on sample size and consistency of findings across multiple studies. Study selection is illustrated in Figure 1.

Overview of childhood myopia

Children with myopia retain near vision; however, distant objects such as classroom boards become blurred. This is due to the excessive axial elongation of the eyeballs, causing incoming light to be focused anterior to the retina rather than directly onto it. This is associated with reduced distance vision [10]. This disease can then progress to high myopia. This is defined as a spherical equivalent of ≤-6.00 dioptres and is associated with axial elongation, which underpins the structural changes responsible for later pathological complications [10].

Genetics, environment and lifestyle are the main risk factors associated with the development and progression of myopia [11]. In addition to these established factors, systemic inflammatory and dietary influences have increasingly been explored in chronic paediatric conditions. Emerging evidence suggests that diet-associated inflammatory burden may contribute to disease modulation in children [12]. Progression rates are impacted by the age of onset, sex and ethnicity, with younger patients demonstrating faster progression and certain ethnic groups exhibiting higher progression rates [5]. Earlier onset of myopia is associated with greater disease progression leading to increased chances that the patient may progress to high myopia in adulthood [2,5]. Research has highlighted that family history is a strong predictor of developing myopia [11]. Children who have an affected parent or parents are more likely to develop myopia themselves [13]. Furthermore, prolonged near-work activities, such as reading and up-close use of devices, have also been linked to deterioration of the condition [14-16]. A strong association has been found between children who spend time outdoors and a decreased risk of developing myopia. Wearable light sensors have confirmed that higher daily light exposure is associated with reduced myopia onset risk [15], supporting the epidemiological findings and reinforcing the point that outdoor light is a protective factor, highlighting the importance of lifestyle and its role as a positive factor [14-16].

## Review

NHS management

Within the NHS, childhood myopia is managed in the community by routine eye examinations, with an emphasis on detecting and correcting refractive error [6,17]. Traditional single vision glasses are the standard method of correction. Contact lenses may be used depending on the child’s age, maturity and clinical indication. Whilst these interventions improve visual acuity, they do not address the underlying pathology or slow the progression of myopia [6].

Lifestyle advice is a common part of the NHS management place, reflecting the growing evidence linking lifestyle, environmental and behavioural factors with myopia development. Families are encouraged to increase children’s outdoor time and reduce excessive close reading and screen time [14-16]. This is backed by epidemiological findings showing an association between outdoor exposure and reduced myopia onset; however, their impact on established progressive myopia is modest [11,14,16]. Consequently, these measures are used as a preventative measure.

The restricted scope of the NHS’s management of myopia is shaped by wider system pressures, funding limitations, appointment durations and the absence of national guidelines for myopia control treatments [6,17]. As a result, evidence-based interventions designed to slow myopia progression are not accessible within the NHS care.

Private management

In the UK’s private sector, myopia management options are both optical and pharmacological.

Optical Interventions

Optical strategies for myopia include contact lenses and glasses. These are designed to alter peripheral retinal defocus, which reduces the stimulation for axial elongation and, therefore, the rate of myopia progression [18]. Dual-focus and multifocal soft contact lenses have been significantly studied, with randomised controlled trials (RCTs) showing reductions in both refractive progression and axial length growth when compared with single vision lenses [8,18,19]. The MiSight one-day dual-focus soft contact lenses are supported by robust RCT evidence demonstrating a significant reduction in axial elongation and myopia progression when compared to single vision lenses [18,19].

Orthokeratology is a widely used optical intervention in myopia management, which involves wearing a rigid gas-permeable contact lens overnight that gently reshapes the cornea [20,21]. This provides temporary refractory correction during the day whilst also inducing peripheral myopic defocus [19]. Multiple studies, including RCTs, cohort studies and meta-analyses, have shown that orthokeratology slows axial elongation compared with traditional single vision glasses [21,22]. Effective results require efficient adherence and high-level lens hygiene due to the small but clinically significant risk of microbial keratitis. Careful patient selection and ongoing monitoring are necessary [19,22]. Although orthokeratology carries a risk of microbial keratitis, incidences are similar to other forms of overnight lens wear when appropriate hygiene protocols are followed [20,23].

For children who are not suited to contact lenses, an alternative is myopia control glasses. These glasses have lenses called defocus incorporated multiple segment (DIMS) lenses and highly aspherical lenslet designs. They have shown significant reductions in myopia progression in trials [24,25]. Despite their less effectiveness, their non-invasive nature and ease of use make them appealing to younger patients, leading to their increasing use by the UK’s private sector [24].

Pharmacological Interventions

Pharmacological treatment is another option for managing childhood myopia in the UK’s private sector.

Low-dose atropine eye drops are the most established pharmacological intervention for myopia control [7,26]. Atropine is a non-selective muscarinic antagonist and has been historically used off-label in the UK for myopia management [26]. In November 2025, the Medicines and Healthcare products Regulatory Agency (MHRA) approved Ryjunea (atropine 0.1 mg/mL) as a management option for myopia. This makes it the first licensed treatment in the UK specifically indicated to target myopia disease progression in children aged 3-14 years [27]. Although the exact mechanism is incompletely understood, evidence suggests that atropine’s action is mediated through retinal signalling pathways and modulation of scleral growth rather than cycloplegia alone [28].

Multiple studies have demonstrated that atropine significantly reduces the rate of progression of low and moderate myopia, with a dose-dependent effect on both refractive error progression and axial elongation [29]. Evidence suggests that lower concentrations of atropine offer the most favourable balance between efficacy and tolerability [30]. Earlier studies demonstrated a significant rebound following treatment cessation [29], whereas the lower concentrations reduced this phenomenon [30,31]. Atropine (0.05%) has shown to provide the most consistent therapeutic effects across studies [29,30]. At lower doses, side effects such as photophobia and near vision blur are uncommon. Optimal dosing strategies and long-term outcome following stopping treatment remain areas of ongoing research [7]. Meta-analysis data suggest that combination therapy using low-dose atropine and orthokeratology could provide the greatest reduction in axial elongation when compared to a single method, but long-term data are limited [7].

Therefore, in the UK’s private sector, low-dose atropine is, therefore, considered an option for children with progressive myopia. In cases where optical interventions have not led to disease control, it is therefore frequently used in combination with other optical strategies to control childhood myopia [32].

Table 1 presents a comparative summary of myopia management options, and Table 2 summarises the key studies included in the present study.

**Table 1:** Comparative summary of myopia management options Table showing a comparative summary of all the interventions in this review for myopia, listing the mechanism, evidence strength, availability in the NHS or private and the limitations. Data derived from references [6,7,17-30]

Management options	Mechanism of action	Evidence strength	Availability in the UK	Limitations
Single vision glasses	Correct refractive error only	Low	NHS and private	No effect on disease progression
Dual-focus contact lenses	Peripheral myopic defocus	High	Private	Cost and lens tolerance
Orthokeratology	Corneal reshaping and defocus	High	Private	Infection risk and adherence
Myopia control glasses	Defocus lens segments	Moderate-high	Private	Variable efficacy
Low-dose atropine	Retinal and scleral signalling	High	Private	Side effects and rebound risk

**Table 2:** Summary of the key studies used, including design, size, intervention and outcome Data derived from references [17,18,20-23,29-32] LAMP: Low-dose Atropine for Myopia Progression, RCT: randomised controlled trial, DIMS: defocus incorporated multiple segment

Study	Design	Sample size	Intervention	Main outcome
Chamberlain et al.	RCT	>100	Dual-focus lenses	Reduced axial elongation
Cho and Cheung	RCT	102	Orthokeratology	Slower myopia progression
Lam et al.	RCT	183	DIMS spectacles	Reduced refractive change
Yam et al. (LAMP)	RCT	438	Low-dose atropine	Dose-dependent efficacy

Discussion

The reviewed literature shows strong and consistent evidence that myopia control strategies slow down the progression of childhood myopia by reducing axial elongation and refractive progression. RCTs evaluating dual-focus soft lenses and orthokeratology have shown significant reductions in axial length growth when matched with single vision correction alone [17,19,22,23]. Similar results are found with DIMS lenses and aspherical lenslet design glasses that showed significant slowing of refractive progression in children [24,25].

Pharmacological interventions with low-dose atropine have shown strong evidence of dose-dependent efficacy. The Low-dose Atropine for Myopia Progression (LAMP) study highlighted that 0.05% atropine concentration is the best at achieving a reduction in axial elongation whilst having a side effect profile that is acceptable [30]. Further studies looking at the benefits over a medium time period also supported the use of low-dose atropine as a management option [31].

Meta-analyses comparing optical interventions with orthokeratology and low-dose atropine showed a comparable efficacy across modalities, but when combined, they provided an improved control of progression [7,31].

Behavioural and environmental methods were compared with optical and pharmacological interventions. Increasing children’s outdoor exposure as a method showed that it does have a role in reducing the onset of myopia; however, it is not effective in limiting progression once myopia has been established [10,13-15]. On the other hand, studies showed that increased near work or screen time has been shown to be linked to myopia development [13,14].

The projected impact of high myopia carries significant implications not only for a patient’s visual outcome but also for healthcare sustainability. The management of sight-threatening complications such as retinal detachment, glaucoma and myopic maculopathy is very financially demanding [3,5]. Therefore, proactive childhood control methods provide a wider cost-saving impact by limiting incidences of advanced disease and its burdens [13-15].

Modelling studies highlight the relationship between worsening dioptres and risk of visual impairment with myopia [4]. Decision support tools based on absolute risk showing the impact of early intervention can help communicate this to patients’ families [4,5].

Whilst the short- to medium-term data demonstrates a reduction in axial elongation, long-term evidence looking at whether these control methods have translated into a reduced rate of adult complications remains limited [11,12].

In the UK, however, myopia progression treatments are mostly limited to the private market, as the NHS service mainly concentrates on refractive correction and lifestyle advice [6,16]. As a result, management of childhood myopia varies significantly depending on location and financial situation [6,17], as families that depend solely on the NHS may not be offered these evidence-based novel treatments, despite the growing evidence supporting their effectiveness in slowing down disease progression.

This disparity highlights systemic issues within the NHS, such as funding limitations, brief appointment times and a lack of clear guidelines for managing childhood myopia, rather than a lack of evidence. This reactive approach prioritises visual function rather than disease modification. This is inadequate for children with rapidly progressing disease who are at risk of sight-threatening ocular complications. A timely and proactive approach to clinical management is therefore vital [6,17].

The recent approval of Ryjunea by MHRA is a positive step in the right direction and an important regulatory development as it may help influence commissioning and clinical adoption within the NHS [27].

A renewed focus on delaying myopia progression by the NHS rather than merely correcting refractive error would be better aligned with current evidence supporting early intervention [4]. Bridging the gap between the NHS and private services could lead to better disease modification. This can be actioned through clear national guidelines, appropriate funding, clinical education and well-defined service pathways promoting consistent and equitable care for all children with myopia, irrespective of postcode and financial situation [3,5].

Limitations

This review has several limitations due to its narrative design. It lacks a formal protocol and systematic review methodology, which may have missed relevant studies and introduced selection bias. A formal quality assessment of the included studies is also missing, so the strength of the evidence should be interpreted with caution. The varying designs, population characteristics, interventions and outcome measures limit direct comparison. Publication bias may have influenced the evidence base, as positive findings are more likely to be published. As with all narrative syntheses, interpretation of findings may be subject to author bias. These limitations should be considered when interpreting the conclusions of this review.

## Conclusions

This review provides strong evidence that myopia control interventions can modify disease and slow down myopia progression. Currently, the NHS management focuses on refractive correction and lifestyle advice, whereas the private sector has optical and pharmacological methods to target disease progression. Developing national guidelines and funded care pathways could help improve equitable access to myopia control and reduce the burden of long-term myopia complications and eye burden in the UK.
